# Aberrant Splicing in Transgenes Containing Introns, Exons, and V5 Epitopes: Lessons from Developing an FSHD Mouse Model Expressing a D4Z4 Repeat with Flanking Genomic Sequences

**DOI:** 10.1371/journal.pone.0118813

**Published:** 2015-03-05

**Authors:** Eugénie Ansseau, Jacqueline S. Domire, Lindsay M. Wallace, Jocelyn O. Eidahl, Susan M. Guckes, Carlee R. Giesige, Nettie K. Pyne, Alexandra Belayew, Scott Q. Harper

**Affiliations:** 1 University of Mons, Research Institute for Health Sciences and Technology, Laboratory of Molecular Biology, Mons, Belgium; 2 Center for Gene Therapy, The Research Institute at Nationwide Children’s Hospital, Columbus, OH, United States of America; 3 Biomedical Sciences Graduate Program, The Ohio State University, Columbus, OH, United States of America; 4 Department of Pediatrics, The Ohio State University College of Medicine, Columbus, OH, United States of America; Florida State University, UNITED STATES

## Abstract

The *DUX4* gene, encoded within D4Z4 repeats on human chromosome 4q35, has recently emerged as a key factor in the pathogenic mechanisms underlying Facioscapulohumeral muscular dystrophy (FSHD). This recognition prompted development of animal models expressing the *DUX4* open reading frame (ORF) alone or embedded within D4Z4 repeats. In the first published model, we used adeno-associated viral vectors (AAV) and strong viral control elements (CMV promoter, SV40 poly A) to demonstrate that the *DUX4* cDNA caused dose-dependent toxicity in mouse muscles. As a follow-up, we designed a second generation of *DUX4*-expressing AAV vectors to more faithfully genocopy the FSHD-permissive D4Z4 repeat region located at 4q35. This new vector (called AAV.D4Z4.V5.pLAM) contained the D4Z4/DUX4 promoter region, a V5 epitope-tagged *DUX4* ORF, and the natural 3’ untranslated region (pLAM) harboring two small introns, *DUX4* exons 2 and 3, and the non-canonical poly A signal required for stabilizing *DUX4* mRNA in FSHD. AAV.D4Z4.V5.pLAM failed to recapitulate the robust pathology of our first generation vectors following delivery to mouse muscle. We found that the DUX4.V5 junction sequence created an unexpected splice donor in the pre-mRNA that was preferentially utilized to remove the V5 coding sequence and *DUX4* stop codon, yielding non-functional DUX4 protein with 55 additional residues on its carboxyl-terminus. Importantly, we further found that aberrant splicing could occur in any expression construct containing a functional splice acceptor and sequences resembling minimal splice donors. Our findings represent an interesting case study with respect to AAV.D4Z4.V5.pLAM, but more broadly serve as a note of caution for designing constructs containing V5 epitope tags and/or transgenes with downstream introns and exons.

## Introduction

**Fig 1:**
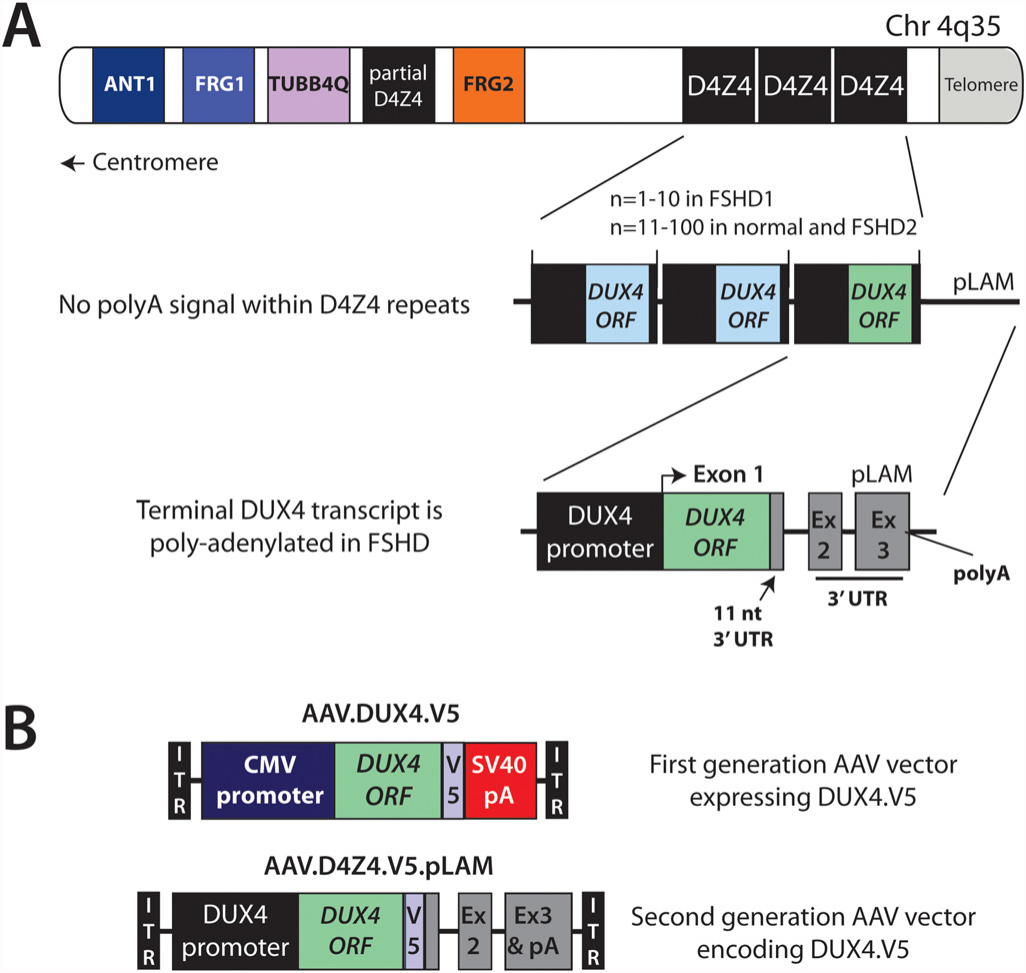
Schematic of chromosome 4, D4Z4, and DUX4-expressing AAV vectors. A: A representation of the telomeric region of the chromosome 4 long arm (4q35). Drawing is not to scale. The 4q35 subtelomere harbors polymorphic, 3.3 kb D4Z4 repeat arrays, as well as other genes, some of which are indicated. This region is normally embedded in repressive heterochromatin. Contraction of the D4Z4 repeat array (in FSHD1) or mutations in SMCHD1 (in FSHD2) leads to epigenetic changes in the 4q35 region, and subsequently permits transcription of the DUX4 gene. An “FSHD permissive” haplotype creates a polyA signal in the pLAM region located downstream of the array. DUX4 transcripts initiated in the last D4Z4 unit extend to this signal and are stabilized by a polyA tail, thereby allowing the mRNA to be translated into the toxic, pro-apoptotic DUX4 protein. B: Two different AAV vectors were engineered to express DUX4. The first generation vector utilized a CMV promoter and SV40 polyA signal. The DUX4 ORF was tagged at the 3’ end with sequences encoding a V5 tag, thereby producing a full-length DUX4 protein containing a carboxy-terminal V5 epitope fusion. ITR, AAV2 inverted terminal repeats. The second generation AAV.D4Z4.V5 vector essentially recapitulates the terminal D4Z4 repeat and pLAM sequences isolated from an FSHD patient, but engineered to express DUX4 with a carboxy-terminal V5 epitope fusion.

Facioscapulohumeral muscular dystrophy (FSHD) is an autosomal dominant muscle disorder characterized by progressive weakness and wasting of specific muscles in the face, shoulder girdle, and limbs [1]. FSHD arises from inappropriate expression of the *DUX4* gene in muscle (Fig. 1A) [2–8]. The *DUX4* open reading frame (ORF) is embedded within each unit of tandemly arrayed DNA macrosatellite sequences, called D4Z4 repeats, on the subtelomeres of chromosomes 4q and 10q. D4Z4 arrays vary in copy number and an individual human genome may contain several hundred virtually identical *DUX4* ORFs. Despite this sequence abundance, FSHD-associated *DUX4* gene expression arises uniquely from the last 4q35 D4Z4 unit, and only when two conditions are met to create a ‘FSHD-permissive’ chromosomal environment. First, the 4q subtelomere must have sufficient euchromatin to allow D4Z4/DUX4 transcription [9–14]. In non-FSHD muscle, 4q35 D4Z4 arrays are hypermethylated and embedded within heterochromatin, thereby suppressing *DUX4* transcription. Contracted D4Z4 arrays in FSHD1 (1–10 repeats on one 4q allele), or mutations in the chromatin modifier gene *SMCHD1* in FSHD2, cause chromatin opening allowing for *DUX4* transcription in FSHD muscle [6,15]. Second, the 4q subtelomere must exist on a specific chromosomal background (4qA) where the terminal D4Z4 unit sits adjacent to a DNA sequence polymorphism harboring two untranslated exons, two small introns, and most critically, a non-canonical poly A signal (called pLAM) required for stabilizing *DUX4* mRNA and permitting translation into toxic, full-length DUX4 protein (Fig. 1A) [3,7,16,17]. FSHD arises only when both conditions are met [6,15].

The current model of FSHD pathogenesis emerged from studies undertaken over the last two decades, but the recognition of DUX4 as a causal factor in FSHD is relatively recent, and has prompted efforts to develop *DUX4*-expressing animal models using transgenics or viral vectors [8,18–20]. In the first published *DUX4* mouse model, we described an adeno-associated viral vector (AAV)-based DUX4 expression system in which a V5 epitope-tagged *DUX4* ORF was driven by the CMV promoter and an SV40 poly A signal (AAV.CMV.DUX4.V5; Fig. 1B) [8,20]. We included the V5-tag because available antibodies were of limited utility for *in vivo* protein detection in mice. This first generation construct produced high levels of functional, full-length DUX4.V5 protein and caused dose-dependent myopathy in mice as early as one-week after injection [8]. This robust system was advantageous because pathology could be expedited to model an otherwise slowly progressive disease, and since we were delivering the *DUX4* ORF without downstream genomic regions, we could focus specifically on the impacts of expressing and inhibiting toxic full-length DUX4 in muscle, while avoiding other non-toxic splice forms which only arise if untranslated exons 2 and 3 (and intervening introns) are present [3,7,8,20,21]. Nevertheless, the widespread and rapid muscle turnover caused by AAV.CMV.DUX4.V5 has not been typically seen in human FSHD patients. We therefore aimed to express *DUX4*.*V5* at more physiologically relevant levels using AAV vectors carrying the *cis*-acting D4Z4, 3’ UTR, and pLAM elements naturally utilized for *DUX4* expression in FSHD-permissive cells (AAV.D4Z4.V5.pLAM; Fig. 1B) [3,6,7]. During the vector development process, we discovered preferential mis-splicing of the *DUX4*.*V5* ORF transcript, facilitated by a splice-donor sequence created at the DUX4-V5 ORF junction and the inclusion of exons and introns in our transgene. We further found that this type of mis-splicing could occur in transgenes expressing other ORFs and downstream genomic sequences. Our findings represent an interesting case study with respect to creating animal models expressing FSHD-permissive genomic elements, but more broadly serve as a note of caution for designing constructs containing V5 epitope coding sequences and/or transgenes with downstream introns and exons.

## Results

Our initial goal was to develop a second-generation AAV vector for expressing *DUX4* in mouse muscle, using natural genomic sequences from the terminal D4Z4 region. These natural sequences included the D4Z4 promoter (which we defined as the first 1.8 kilobases of the D4Z4 repeat), DUX4 exon 1 (the DUX4 ORF with an 11 nucleotide 3’ UTR), and intron 1 from the D4Z4 unit, followed by a pLAM fragment that extended the 3’ UTR with *DUX4* exon 2, intron 2, and exon 3. Importantly, exon 3 contains the non-canonical pLAM poly A (ATTAAA) allowing for DUX4 mRNA stabilization and protein expression in FSHD-permissive myonuclei [3,6]. In addition, we modified the 3’ end of the *DUX4* ORF so the full-length DUX4 protein would include a V5-epitope tag fused to the carboxyl-terminus, as we did with the first-generation CMV-based system we previously described (Fig. 1B) [8,20]. We called the new, second-generation vector AAV.D4Z4.V5.pLAM (Fig. 1B).

**Fig 2:**
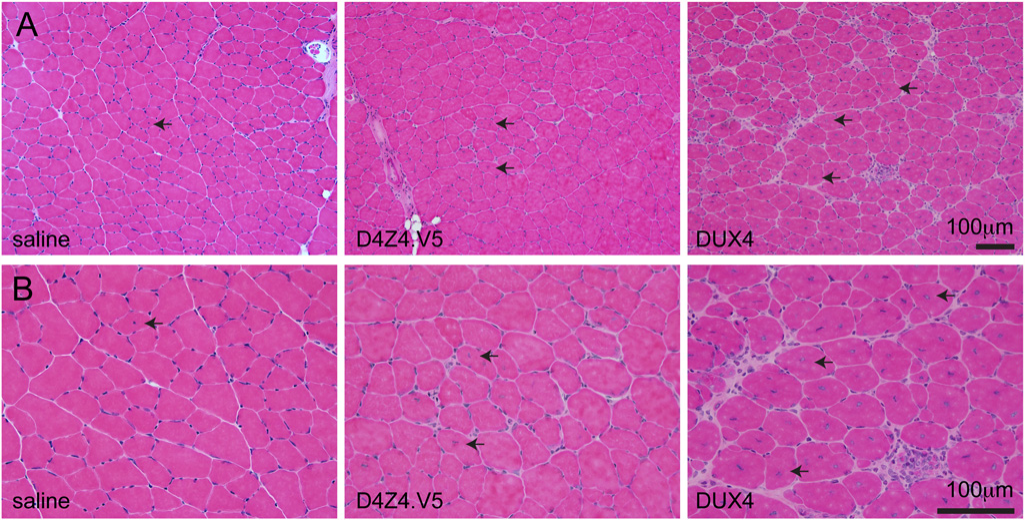
AAV.D4Z4.V5 vectors are non-toxic compared to AAV.CMV.DUX4.V5. H&E stained cryosections of tibialis anterior muscles isolated from C57BL/6 mice, 2 weeks after injection with 3x10^10^ particles of the indicated AAV6 vectors, or saline. Top panels (A) show low power images, and (B) are high power images of the same sections. Arrows point out examples of myofibers with centrally located nuclei, which are a histological indication that muscles were damaged and subsequently repaired. Note that AAV.DUX4.V5 vectors cause widespread muscle damage and regeneration, while AAV.D4Z4.V5 and saline did not.

To test vector potency, we packaged the D4Z4.V5.pLAM provirus into AAV serotype 6 (AAV6) capsids [22], and injected 3 x 10^10^ particles (reported as DNAse resistant particles, or DRP) into tibialis anterior (TA) muscles of wild-type C57BL/6 mice. We also delivered 3 x 10^10^ particles of AAV.CMV.GFP vectors [23], or saline, in separate animals to serve as injection controls. In contrast to the massive muscle lesions and subsequent widespread regeneration induced by CMV-based *DUX4* vectors (Fig. 2) [8], the AAV.D4Z4.V5.pLAM injections failed to cause substantial muscle damage, although a few regenerated myofibers with centrally located nuclei were present (Fig. 2). Using two different V5-specific antibodies, we were unable to detect protein expression by western blot or immunofluorescence (data not shown).

**Fig 3:**
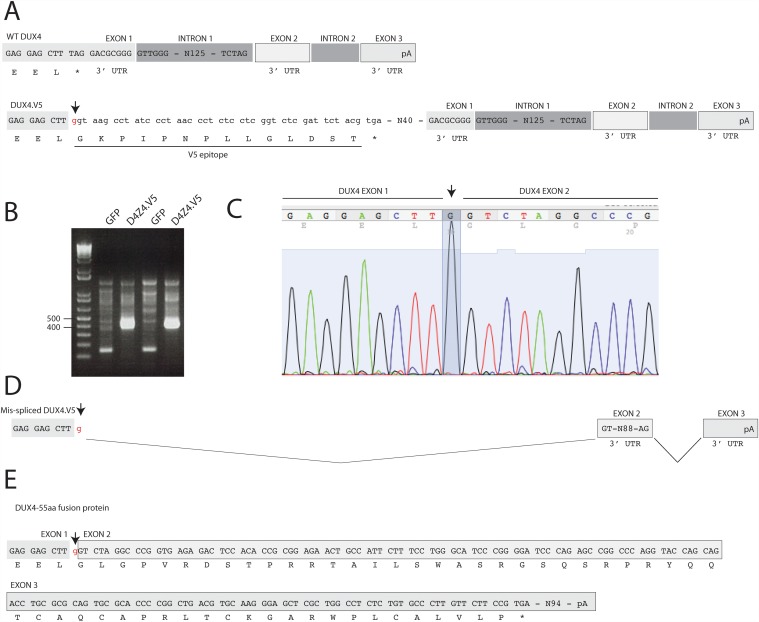
The V5 epitope tag coding sequence caused mis-splicing of DUX4 mRNA. A: *Top* shows the exon-intron structure of the DUX4 gene arising from the distal D4Z4 unit, and flanking pLAM region. Exons and introns are indicated but not to scale. The asterisk (*) represents the normal DUX4 stop codon. The final 11 nucleotides of exon 1 are untranslated, as are those in exons 2 and 3 (the latter is part of the pLAM sequence). In an FSHD permissive genotype, the DUX4 polyA signal is located in exon 3. *Bottom* shows the same locus but with the V5 coding sequences inserted. A new stop codon was placed downstream of the V5 tag, followed by 40 nucleotides of linker sequences (N40). The lower case “g” indicated by an arrow and red text, is the aberrant splice site created by the DUX4-V5 fusion coding sequence. B: AAV.D4Z4.V5 vectors express DUX4.V5 mRNA. A previously reported nested PCR/3’ RACE strategy [24] was used to amplify 3’ end of the DUX4 transcript from RNAs harvested from AAV.D4Z4.V5-injected C57BL/6 TA muscles. A ~450 bp band was amplified from muscles that received AAV.D4Z4.V5 but not AAV.GFP controls. The size of this band corresponded to the expected product of the reverse transcribed and PCR-amplified DUX4 3’ end if introns 1 and 2 were spliced out. C: Sequence chromatogram of cloned 3’ RACE products identifying the mis-splicing event. The arrow points out the same g nucleotide indicated in panel A. D: The V5 epitope, N40 linker sequences, and intron 1 are spliced out of the D4Z4.V5 transcript. E: This mis-splicing event created a new missense DUX4 protein containing an additional 55 amino acids (DUX4-55aa), until an in-frame stop codon occurred in exon 3.

**Fig 4:**
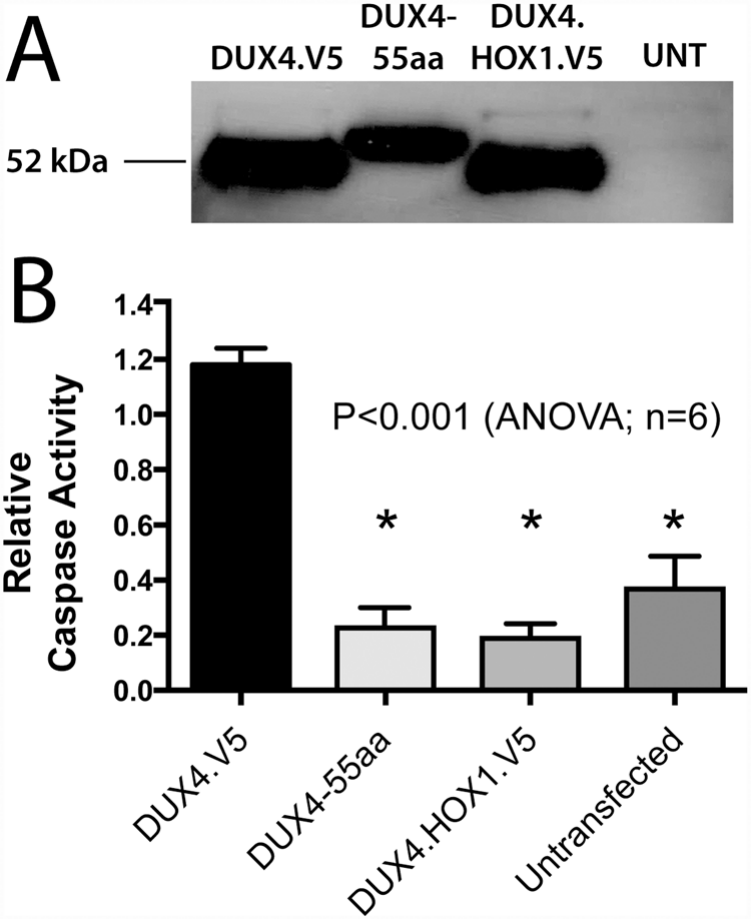
The DUX4-55aa protein expressed from mis-spliced DUX4 V5 mRNA does not activate apoptosis in vitro. (A) Western blot confirms expression of DUX4.V5, DUX4-55aa, and DUX4.HOX1.V5 constructs in HEK293 cells transfected two days earlier. The DUX4-55aa construct lacked a V5 tag, and proteins were therefore detected using rabbit anti-DUX4 primary antibodies followed by HRP-coupled goat-anti-rabbit secondary antibodies. DUX4-55aa is ~6 kDa larger than full-length DUX4, which migrates at ~52 kDa. (B) DUX4-55aa protein does not activate apoptosis in vitro. HEK293 cells were transfected with 200 ng of the indicated CMV expression plasmids and plated simultaneously on 96-well plates. Caspase-3/7 activity was measured 48 hours later using a fluorescent plate reader. High caspase-3/7 activity in CMV.DUX4.V5-transfected HEK293 cells indicated that the DUX4.V5 protein caused apoptotic cell death, as previously reported [8]. The non-toxic DUX4.HOX1.V5 protein, which was engineered to lack DNA binding function, failed to induce apoptosis, as expected [8]. Likewise, CMV.DUX4-55aa did not activate caspase-3/7. *, indicates significant difference from DUX4.V5-treated cells. Data represent means +/- sem, averaged from 6 separate samples performed from two independent experiments. UNT, untransfected.

We next turned to a more sensitive detection method and examined *DUX4* mRNA expression in AAV.D4Z4.V5.pLAM-injected TA muscles. We extracted total RNA from injected animals, generated cDNA by reverse transcription with an oligo dT primer containing a 3’ adaptor, and amplified *DUX4* cDNA sequences using an established 3’ RACE/nested PCR strategy [24]. We amplified a ~450 base pair (bp) DNA fragment from muscles injected with AAV.D4Z4.V5.pLAM, which was expected if introns 1 and 2 had been spliced out from the *DUX4* mRNA (Fig. 1 and Fig. 3) [7]. Importantly, this band was absent in AAV.CMV.GFP-treated controls. DNA sequencing confirmed that the ~450 bp product indeed included full-length *DUX4* lacking introns 1 and 2, however the V5 epitope-encoding sequences and stop codon were absent (Fig. 3A, C-D). Instead, the cDNA product contained an unexpected fusion of the full-length *DUX4* ORF 3’ end (CTT codon, which encodes Leu424 in the full-length protein; Fig. 3) to *DUX4* exons 2 and 3, which are normally untranslated [7]. Another in-frame stop codon was reached 168 nucleotides downstream in exon 3 (Fig. 3). This mis-spliced transcript was translated into an unnatural, 479 amino acid protein composed of full-length DUX4 with 55 missense amino acids fused to the carboxyl-terminus (DUX4-55aa; Fig. 3). The 55 additional amino acids negatively affected the previously described pro-apoptotic function of DUX4, as the DUX4-55aa protein failed to activate caspase-3/7 in transfected cells (Fig. 4) [2,3,5,7,8,24,25].

**Fig 5:**
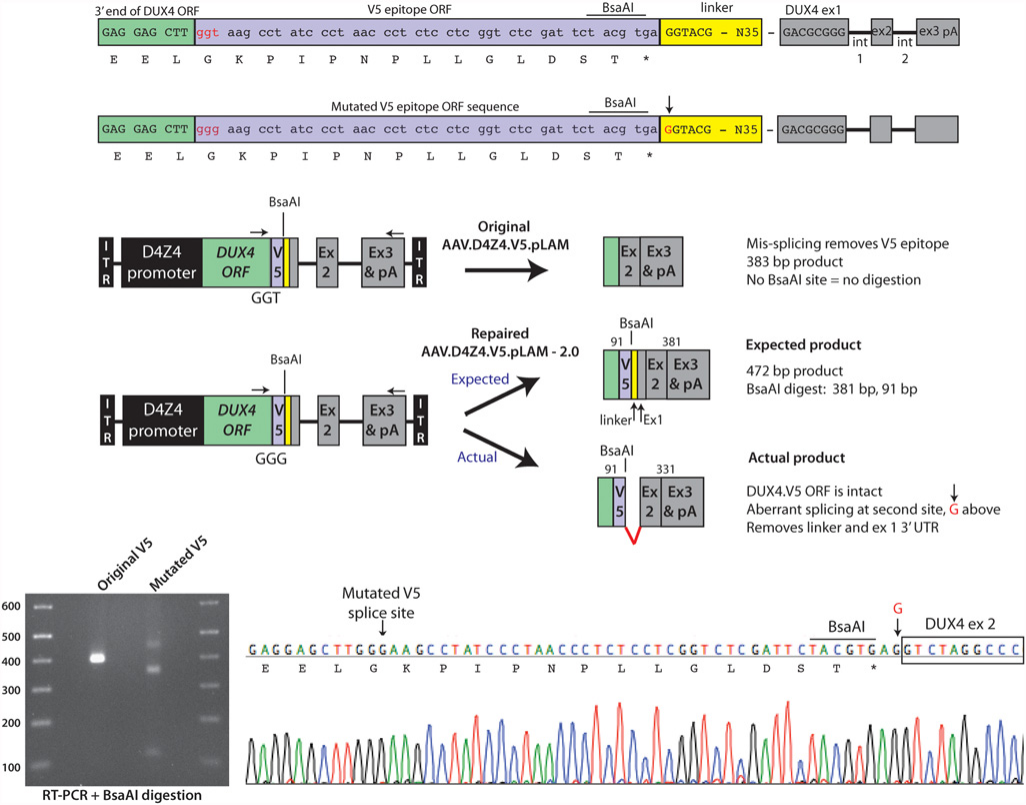
Repaired AAV.D4Z4.V5.pLAM-2.0 vector produces full-length DUX4.V5. (A) Mutation of the DUX4-V5 splice donor from GGT to GGG (lowercase red letters) maintained the V5 glycine residue but destroyed the invariant T required for splicing. Note the yellow boxed area containing linker sequences joining the V5 epitope to the natural DUX4 exon 1 untranslated region. The red uppercase G (indicated by arrow) at the beginning of the linker indicates a second aberrant splice site utilized instead of the natural DUX4 exon 1 splice donor, only in the repaired sequence. BsaAI restriction sites are indicated. (B) Schematic of 3’ RACE/BsaAI restriction digestion assay to identify mis-spliced DUX4-V5 products. The BsaAI site is located in the V5 tag. Removal of this sequence by mis-splicing creates a BsaAI-resistant 3’ RACE product of 383 bp, evident by gel electrophoresis (C, original V5). The repaired vector incorporated the V5 tag and its resident BsaAI, making this product susceptible to digestion by the enzyme. The expected full-length and BsaAI-digested products were empirically smaller following electrophoresis (C, mutated V5). (D) Sequence chromatogram of the mutated DUX4.V5 transcript revealed that full-length DUX4.V5 was produced, but a second splice donor sequence was encountered and preferentially utilized instead of the natural DUX4 exon 1 donor. Arrow points to the G residue indicated in the linker sequence of (A).

Close examination of the DUX4 ORF-exon 2 junction (DUX4-55aa encoding sequence) suggested a mechanism for the unexpected mis-splicing. The Life Technologies/Invitrogen V5-encoding sequences, when fused in frame with the 3’ end of the *DUX4* ORF, created a functional splice donor site at the DUX4-V5 ORF junction (Fig. 3). Specifically, the site created by the *DUX4-V5* fusion was 5’ -TTGGTAAG—3’, while the consensus minimal splice donor sequence is 5’—(A/C)AGGTAAG—3’ (Fig. 3) [26]. Because this unintended splice site rendered our AAV.D4Z4.V5.pLAM vector useless for expressing functional DUX4.V5 protein, repair was required. To do this, we mutated the wobble position of the first glycine codon of the V5 ORF from GGT to GGG, which also encodes glycine (Fig. 5). The GT from the original GGT codon sequence, when placed in context with the 3’ end of DUX4 ORF, constituted a core splice donor sequence, while the GG in the new GGG codon was not a splice site (Fig. 3 and Fig. 5) [26]. We next injected mouse TA muscles with 3 x 10^10^ DRP of the repaired vector (called AAV.D4Z4.V5.pLAM-2.0), and the original AAV.D4Z4.V5.pLAM. We then harvested mRNA, amplified the DUX4 mRNA using 3’ RACE, and examined the spliced cDNAs using diagnostic restriction digestion and DNA sequencing.

For the restriction digestion assay, we took advantage of a BsaAI cut site in the V5 epitope coding sequence (5’ -TACGTG—3’), which was absent in mis-spliced *DUX4* transcripts (Fig. 5). The resulting 3’ RACE product would therefore be resistant to BsaAI and migrate as a single band on an agarose gel. Indeed, we found that the original AAV.D4Z4.V5.pLAM vector exclusively produced a BsaAI-resistant product that migrated around the expected ~383 bp size (Fig. 5). We also directly sequenced the 3’ RACE products, and never found evidence of other splicing events (based on the absence of mixed peaks in chromatograms; data not shown). For the repaired vector, we expected correctly spliced *DUX4-V5* mRNA to yield a 472 bp 3’ RACE product containing a BsaAI site allowing for cleavage into two smaller fragments (381 bp and 91 bp). Indeed, a full-length product migrated in the agarose gel around the expected location, and was cleaved into two smaller products by BsaAI. The larger of these two BsaAI products (381 bp) migrated as expected, while the smaller ran slightly slower than predicted (91 bp) (Fig. 5). Cloning and sequencing the full-length product revealed that our splice-site mutagenesis strategy worked as intended, as the second-generation AAV.D4Z4.V5.pLAM-2.0 vector produced a DUX4.V5 mRNA that was not mis-spliced at the DUX4-V5 junction (Fig. 3 and Fig. 5). Indeed, this transcript yielded a full-length *DUX4* ORF fused in-frame with the V5 epitope ORF, and had a correctly localized stop codon. Nevertheless, this transcript was also incorrectly spliced at a site just downstream of the V5 stop codon, in a linker region located 47 nt before the natural 3’ end of DUX4 exon 1 (Fig. 3 and Fig. 5).

The discovery of two unexpected splice donors in our original and repaired AAV.D4Z4.V5.pLAM vectors spurred us to investigate the potential for mis-splicing of intron-containing transgenes in other contexts, using several approaches. We first performed a bioinformatics analysis to predict the number of cDNAs susceptible to mis-splicing when fused at the 3’ end with V5 encoding sequences and placed in the same genomic context we used for *DUX4*. To do this, we downloaded 29,064 human and 23,089 mouse transcripts deposited in CCDS libraries at NCBI (ftp://ftp.ncbi.nlm.nih.gov/pub/CCDS), removed the stop codons, and fused the 3’ end of each transcript in frame with the Life Technologies/Invitrogen V5 coding sequence. We then searched for junction sequences that created consensus minimal splice donor sites (5’—(A/C)AGGTAAG—3’) and those with the “DUX4 site” (5’ -TTGGTAAG—3’) [26]. We estimated that 3,444 human (11.8%) and 2,623 mouse (11.4%) cDNAs would be potentially mis-spliced using the consensus donor sequence, with another 1,609 human (5.5%) and 1,162 mouse (5.0%) transcripts containing the imperfect “DUX4 site” we reported here (S1 Table).

**Fig 6:**
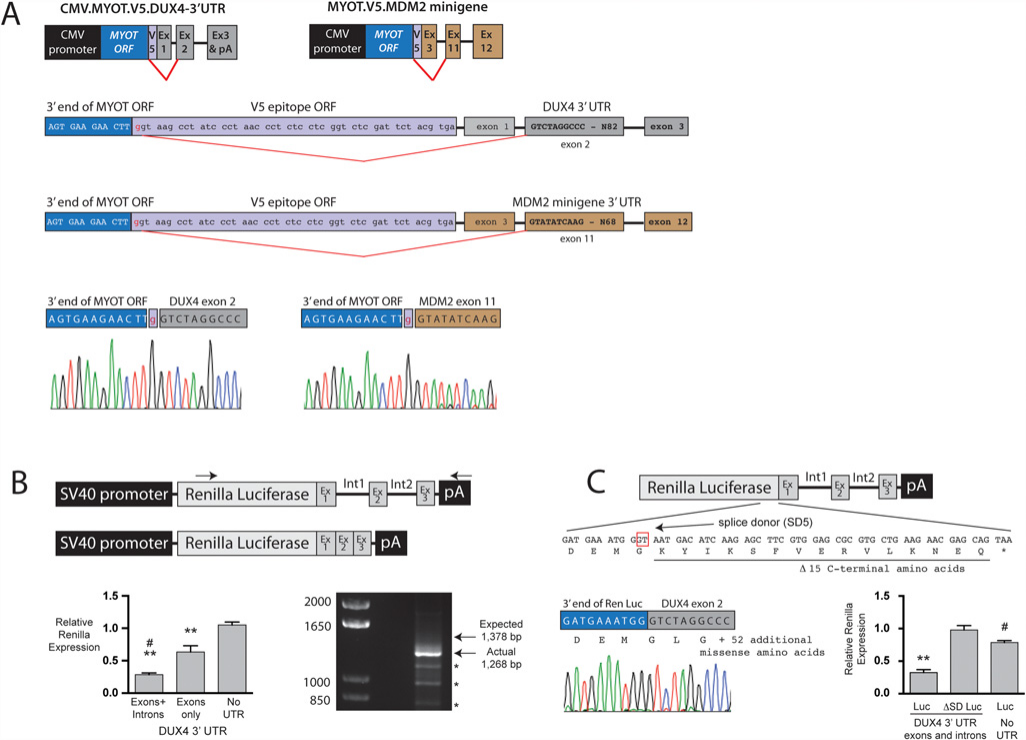
Mis-splicing is not unique to DUX4.V5 cDNAs. (A) The myotilin cDNA fused to V5 creates a functional splice donor that was fused to splice acceptors resident in the DUX4 3’ UTR and that of an unrelated gene, MDM2. In both circumstances, sequence chromatograms showed that the V5 tag was deleted, similar to the event that occurred in the original DUX4.V5 transgene arising from the AAV D4Z4.V5.pLAM vector. (B) The humanized renilla luciferase cDNA contained 5 predicted splice donor sites. When attached to the DUX4 3’ region (exon 1 UTR, intron 1, exon 2, intron 2, exon 3), one site (SD5; boxed in red) was utilized and fused to DUX4 exon 2. PCR of 3’ RACE products produced the SD5 transcript (1,268 bp) and some smaller products on an ethidium bromide stained gel. Only the 1,268 bp band contained Renilla luciferase sequences, and chromatograms confirmed the SD5-mediated mis-splicing event. This construct showed significantly reduced luciferase enzyme activity in vitro (indicated by **, p<0.0001; ANOVA with Tukey’s multiple comparison test, n = 3 replicates). Mutating the SD5 T nucleotide (boxed) destroyed the SD5 splice donor and restored luciferase activity to above normal levels (indicated by #, p = 0.0076; ANOVA with Tukey’s multiple comparison test, n = 3 replicates). (C) Comparison of splice donor sequences identified in this study to the consensus splice donor site.

Next, we sought to empirically confirm mis-splicing in another V5-tagged gene from the list generated above. For this, we chose *myotilin* (*MYOT*) because we had previous experience working with the gene and already possessed *MYOT* expression constructs in the lab. Similar to the *DUX4*.*V5* expression cassette in the first generation D4Z4.V5.pLAM vector, we modified the 3’ end of a CMV promoter-driven *MYOT* cDNA to include the V5 ORF and *DUX4* exons and introns (Fig. 6A). We also generated a second construct containing a *MYOT*.*V5* fusion and genomic elements from the *MDM2* gene as a control [27], to determine if the mis-splicing event was uniquely dependent upon *DUX4* exons and introns, or not (Fig. 6A). We then transfected HEK293 and C2C12 cells with CMV.MYOT.V5.DUX4-3’ UTR or CMV.MYOT.V5.MDM2-3’ UTR plasmids, harvested RNA, and generated 3’ RACE products that were cloned and sequenced, as previously done for *DUX4*. As predicted, we found that the *MYOT-V5* ORF fusion was aberrantly spliced precisely like the *DUX4-V5* ORF arising from the original AAV.D4Z4.V5.pLAM vector, such that the V5 coding sequence was excised and *DUX4* exon 2 was fused to the 3’ end of the *MYOT* ORF (Fig. 6A). Moreover, this aberrant splicing occurred in the *MYOT* transgene containing the *MDM2* 3’ UTR as well, demonstrating it did not uniquely require *DUX4* exons and introns (Fig. 6A).

We next investigated the potential for aberrant splicing of natural, untagged cDNAs when placed in context with 3’ genomic elements. To do this, we inserted the 3’ UTR regions of *DUX4* (the last 11 bp of DUX4 exon 1, intron 1, exon 2, intron 2, and exon 3) downstream of an SV40 promoter-driven *Renilla reniformis* luciferase expression construct [28]. We again transfected HEK293 cells, harvested RNA, and performed 3’ RACE using primers spanning the entire Luciferase-DUX4 3’ UTR transcript (Fig. 6B). Following electrophoresis, we noted one major DNA band that migrated ~100 bp faster on an agarose gel than the expected full-length product, and 3 smaller distinct bands of lesser intensity (Fig. 6F). We gel-purified and cloned each product, then sequenced 5 colonies containing the appropriately sized inserts from each band. The three smaller products were PCR artifacts unrelated to *luciferase* and *DUX4*. However, the most intense 1,268 bp product contained the *Renilla luciferase* ORF lacking the last 50 bp, fused to *DUX4* exon 2 and 3. The resultant transcript produced *Renilla* luciferase protein minus the last 15 carboxyl-terminal residues, fused to an unnatural 52 amino acid peptide translated from *DUX4* exons 2 and 3 (Fig. 6B). This mutation significantly reduced *Renilla* luciferase activity 2.5-fold *in vitro* compared to an identical *Renilla* luciferase expressed from a cDNA lacking downstream exons and introns (Fig. 6B). Importantly, mutating the splice site restored expression of luciferase with activity at or above normal levels (Fig. 6B).

## Discussion

We began this project with the goal of modeling FSHD in mice, but because of splicing artifacts in our DUX4.V5 AAV expression vectors, this report is more focused on the molecular biology of transgene development and vector optimization following incorporation of genomic elements (introns and exons) and a commonly used V5 epitope ORF in our transgene constructs. Importantly, we found that aberrant splicing was not unique to our D4Z4.V5.pLAM system alone, and any cDNA expression system could be affected in a similar fashion if the right genomic context was provided. Thus, considering these defects negatively affected progress and financial resources, we felt it was important to describe and publish our findings as a cautionary note for others designing similar gene expression tools. Below we discuss how our findings from the D4Z4.V5.pLAM system could generally impact other systems.

Our strategy for developing FSHD mouse models involved creating AAV vectors expressing only the DUX4 ORF (exon 1) [8], or the DUX4 ORF containing the natural DUX4 3’ UTR (exons 2 and 3 with the DUX4 poly A signal) and intervening introns (an FSHD-permissive locus). In each case, we added V5-epitope encoding sequences to the DUX4 3’ ORF to facilitate protein detection in mice. V5 had been routinely used in published studies and was a major component of a large line of commercially available expression vectors (Life Technologies/Invitrogen) [29,30]. Moreover, we had prior success tagging and detecting proteins with the V5 epitope and antibodies, and found that DUX4 pro-apoptotic function was unaffected by V5 modification *in vitro* and *in vivo* [8].

In our first published study, using AAV vectors and viral regulatory elements (CMV promoter, SV40 poly A) to express only the DUX4.V5 ORF in mouse muscle, we detected full-length DUX4.V5 mRNA, DUX4.V5 protein, and dose-dependent toxicity in mouse muscles [8]. Although the splice donor sequence created by DUX4-V5 ORF fusion (Fig. 3) was present in this vector, we never noted any unexpected splicing abnormalities. In contrast, when we delivered vectors containing the identical *DUX4. V5* DNA sequence positioned in a genomic context (i.e. upstream of *DUX4* exons and introns), we observed virtually quantitative mis-splicing in the resulting mRNA, between the DUX4-V5 junction splice donor site and the natural *DUX4* intron 1-exon 2 splice acceptor, resulting in V5 tag deletion and production of the longer, non-toxic DUX4-55aa protein (Figs. 3, 4 and 5). The major difference here was that the first generation CMV vector lacked resident splice acceptors (i.e. no 3’ exons and introns) while the second-generation vector contained them. These results demonstrated that the V5 coding sequence had the potential to create an excellent splice donor if fused to the right cDNA sequence, but the site was only utilized when a downstream splice acceptor was provided.

This finding leads to a larger point. Gene expression vectors, including plasmids, viral vectors, or germline transgenes, have been historically designed to express only a cDNA, and often if an intron is included it is located between the promoter and translation start codon. Thus, some transgenes containing sequences resembling a minimal splice donor could be prone to unanticipated aberrant splicing if located upstream of a functional splice acceptor. Indeed, we showed that the *Renilla luciferase* ORF sequence contained a non-consensus but functional splice donor (SD5) that was preferentially spliced to DUX4 exon 2 when the DUX4 3’ UTR was included in the vector (Fig. 6B). Interestingly, this site was predicted in an online splice-site search engine (NNSPLICE0.9; Neural Network) [31], suggesting that potential mis-splicing events can be anticipated. However, four additional sites were also predicted but we never detected those splice forms. Regardless, splicing involving the SD5 site resulted in luciferase protein with significantly reduced activity, which could be restored with targeted mutation of the splice donor (Fig. 6B). We therefore showed that aberrant splicing could cause expression of two different functionally inactivated proteins (DUX4-55aa and *Renilla* luciferase SD5). In addition, we detected a similar mis-splicing event in the *myotilin* pre-mRNA, using two different downstream splice acceptor sequences, giving rise to an abnormal MYOT protein. These results support that transgene mis-splicing could potentially occur in any expression construct under specific conditions (i.e. minimal, non-consensus splice donor; downstream splice acceptor). Indeed, even vectors lacking downstream exons and introns could conceivably be aberrantly spliced. For example, integrating lentiviral vectors or transgenic mice containing random cDNA insertions could produce improperly spliced RNAs if the expression construct integrated near a natural exon-intron boundary.

Our data show that transgene mis-splicing events, such as those described here, can be prevented or minimized. For example, we mutated the *DUX4-V5* junction in our first generation D4Z4 vector, generating AAV.D4Z4.V5.pLAM-2.0 (Fig. 5). This change effectively prevented mis-splicing and mRNAs with the full-length *DUX4. V5* ORF were produced (Figs. 3, 5). Similarly, mutation of the SD5 splice donor in *Renilla* luciferase restored luciferase expression with activity to above wild-type levels, again demonstrating that vectors can be optimized with simple nucleotide changes (Fig. 6). Indeed, sequence optimization of transgenes is already commonly practiced today, as many foreign transgenes are now “codon-optimized” to facilitate more efficient translation. In a like manner, transgenes could also be optimized to avoid mis-splicing.

Nevertheless, proactively mutating potential splice donors requires some ability to predict those with potential function. Here, we noted the difficulties involved in accurately accomplishing this task. For example, none of the splice sites we empirically identified in this study have 100% homology with the consensus minimal splice donor sequence (Fig. 6) [26]. Indeed, among those located in our *DUX4. V5* constructs, the natural *DUX4* exon 1-intron 1 splice donor is weakest, while the original *DUX4. V5* presents the strongest splice site based on sequence. It is therefore possible that mis-splicing events in our constructs might not be as prevalent if the natural *DUX4* exon 1 splice donor was stronger (Fig. 6). As another example of the difficulties inherent in predicting functional splice donors, we noted that only 1 of 5 predicted internal donor sites in *Renilla* luciferase was spliced to DUX4 exon 2 (Fig. 6). Finally, we retrospectively found the *DUX4-V5* junction site predicted as 1 of 24 potential splice donors in the original *DUX4. V5* sequence by the Human Splicing Finder tool, although we found no evidence that the others were functional [32]. Thus, when expressing a cDNA containing downstream genomic elements, or before investing time and money to generate transgenic animals where a cDNA may be incorporated near a natural intron-exon boundary, it may be prudent to empirically test the potential for aberrant splicing using methods similar to those we employed in this study.

## Materials and Methods

### Construction of expression plasmids and AAV proviruses, and AAV6 vector production

Five different AAV vectors were used in this study. All were packaged with AAV6 capsids as previously described [8]. Vectors carrying CMV.eGFP, CMV.DUX4.V5 and CMV.DUX4.HOX.V5 expression cassettes were previously described [8,23]. The D4Z4.V5 and D4Z4.V5.2 vectors were newly constructed for this work. FSHD-associated D4Z4 sequences were originally derived from a pGEM clone containing a 13,477 bp insert isolated from an FSHD patient (GenBank accession AF117653.2) [33]. This clone included 2.5 D4Z4 copies and the pLAM region (with DUX4 untranslated exons 2 and 3 and a DUX4 mRNA-stabilizing polyA signal) situated downstream of the last repeat (pLAM region) [33].

#### AAV.D4Z4.V5 construction

We began construction of AAV.D4Z4.V5 using our previously described pAAV.CMV.hrGFP construct as a base plasmid [34]. This plasmid contains AAV2 ITRs flanking the insert DNA. The insert can be completely removed with Not I restriction digestion, but re-ligation of the plasmid, which would result in placing the two AAV ITRs adjacent to one another, proved problematic. We therefore amplified a stuffer DNA sequence (the hrGFP open reading frame) by PCR using a 5’ primer containing 5’ *NotI*—*KpnI* restriction sites and a 3’ primer containing 5’ *NotI—NcoI* restriction sites. We then ligated this stuffer sequence into the AAV2 backbone plasmid using *NotI* sites, creating an intermediate pAAV.linker plasmid. In the next step, we ligated the terminal D4Z4 repeat from the pGEM clone into the *KpnI* site we created just downstream of the 5’ *NotI* site in the pAAV.linker plasmid, to create a new subclone called pAAV.D4Z4.linker. This construct contained an entire D4Z4 repeat upstream of the hrGFP open reading frame. Next, in a separate subclone, we used recombinant PCR to add the pLAM sequences to the 3’ end of our DUX4.V5 construct, creating pTOPO-Blunt.DUX4.V5.pLAM. An *NcoI* site was added to the 5’ end of the reverse primer we used to amplify the pLAM region. We then digested the pTOPO-Blunt.DUX4.V5.pLAM plasmid with *Bsu36I* (which cuts within the DUX4 ORF) and *NcoI* (added to the end of pLAM), and cloned this 1,449 bp band (encompassing part of DUX4, the V5 tag, and pLAM region) into pAAV.D4Z4.linker digested with *Bsu36I* and *NcoI* restriction sites. This series of digestions and ligations removed the 3’ end of the D4Z4 element and the hrGFP linker sequences from pAAV.D4Z4.linker, and replaced them with the identical D4Z4 sequences downstream of *Bsu36I*, plus the V5-epitope tag (fused to the DUX4 ORF 3’ end), and pLAM. The resultant AAV.D4Z4.V5 plasmid contained a 4,001 bp insert cloned between the two 145 bp (each) AAV2 ITRs. The total genome size of the final vector was therefore 4,291 nucleotides.

#### AAV.D4Z4.V5.2 construction

The V5 glycine codon (GGT), which harbored the D4Z4.V5 splice site identified in this paper, was mutated to a GGG by recombinant PCR using the pTOPO-Blunt.DUX4.V5.pLAM as a template plasmid. This repaired product contained the 3’ end of DUX4 ORF (including the *Bsu36I* site), a new V5 tag, and the pLAM sequences) and was subsequently subcloned into a pBlunt-II PCR cloning vector and sequence verified. The new subclone was then inserted into pD4Z4.V5 as a *Bsu36I* and *NcoI* fragment (as described above), to create pD4Z4.V5.2, which was also 4,291 nucleotides in size as an AAV vector.

#### *Renilla* Luciferase plasmids

The DUX4 3’ UTR region (including the 11 untranslated bp from exon 1, intron 1, exon 2, intron 2, and exon 3) was PCR amplified and cloned into a pENTR-TOPO (Invitrogen), sequence verified, and then inserted by Gateway LR recombination (Invitrogen) into the psiCHECK-DEST vector we previously described [28]. The resultant product contained SV40 promoter-driven *Renilla luciferase* with the *DUX4* 3’ UTR inserted after the stop codon, and separate thymidine kinase (TK) promoter-driven Firefly *luciferase* to serve as a transfection control. Recombinant PCR was used to create the *Renilla luciferase* SD5 mutant. PCR was performed with a forward primer (5’-aaggagaagggcgaggttagac-3’) located in the *Renilla luciferase* coding region, and a reverse mutagenesis primer containing a 5’ SgfI restriction site and the SD5 splice site substitution (5’- CTCGAGCGATCGCCTAGAATTACTGCTCGTTCTTCAGCACGCGCTCCACGAAGCTCTTGATGTACTTCCCCATTTCATCTGGAGCGTC-3’). The resulting 340 bp band was digested with SgfI and AatII, yielding 79 bp and 249 bp bands. The latter product was ligated into the original *Renilla luciferase-DUX4* 3’ UTR plasmid using the same enzyme sites to create the final SD5 mutant.

#### Myotilin plasmids

To construct the *myotilin* plasmids, we used PCR to add both a V5 epitope tag and flanking NheI and Acc65I restriction sites to the *MYOT* ORF (forward 5’-AAAAGCTAGCCCACCATGGCTCGCAGATTGCTAGG-3’ and reverse 5’-TTTTGGTACCCTACGTAGAATCGAGACCGAGGAGAGGGTTAGGGATAGGCTTACCAAGTTCTTCACTTTCATAGAGTC-3’). This fragment was sequence verified and subcloned into our AAV.CMV expression vector. To add the terminal region of the D4Z4 repeat, we amplified regions of the PGEM42 vector with primers beginning at the untranslated region of exon 1 of DUX4 and terminating at the end of exon 3 flanked by BsiWI restriction sites (sense 5’-TTTTCGTACGTAGGACGCGGGGTTGGG-3’ and antisense 5’-TTTTCGTACGGGATCCGGGAGGGGGCATTTTAATATATCTCTG-3’[33]. After restriction enzyme digestion, BsiWI overhangs are compatible with the Acc65I overhangs at the 5’ end of the *MYOT. V5* ORF. The *MDM2* sequences were added by amplifying the 3–11–12 MDM2 minigene [27] with primers beginning at exon 3 and terminating at the end of exon 12 flanked by Acc651 restriction sites to insert the fragment at the end of the MYOT.V5 ORF (forward 5’-AAAAGGTACCGAGCTCTGTACCTACTGATGG-3’ and reverse 5’-TTTTGGTACCGCCTCAACACATGACTCTCTG-3’).

### Luciferase Assay

The terminal region of the D4Z4 repeat was cloned downstream of the *Renilla luciferase* stop codon, thereby functioning as a 3′ UTR. A separate thymidine kinase (TK) promoter-driven Firefly *luciferase* cassette, present on the same plasmid, served as a transfection control. HEK293 cells were cotransfected in triplicate wells (Lipofectamine-2000; Invitrogen, Carlsbad, CA) with 200 ng of the *luciferase* DUX4-3’UTR reporter according to manufacturer’s instructions. Luciferase activity was determined by measuring *Renilla* and Firefly luciferase activity (Dual Luciferase Reporter Assay System; Promega) 24 hours post-transfection following manufacturer’s instructions. Triplicate data were averaged and the results were reported as the mean ratio of *Renilla* to Firefly activity ± SEM.

### Intramuscular injections of AAV vectors in mice

Ethics Statement: The Institutional Animal Care and Use Committee (IACUC) at the Research Institute at Nationwide Children’s Hospital (IACUC Number: AR13-00016; SQH, PI), approved this study, and all animals were housed and handled according to IACUC guidelines.

For injections, adult C57BL/6 mice received a 50 microliter volume containing 3x10^10^ DNAse resistant particles (DRP) of AAV6 carrying the respective vector genomes described above, or saline. Animals were injected bilaterally in the tibialis anterior (TA) muscle. Muscles were harvested for subsequent analysis ~2 weeks after vector delivery. Muscle cryosections were stained by H&E as previously described [8].

### RNA isolation and 3′ RACE

Total RNA was extracted from pulverized, flash frozen TA muscles using Trizol Reagent (Life Technologies), following manufacturer’s protocol. Reverse transcription was performed on 0.5 μg of DNase-treated RNA with the 3′ adaptor of the RLM-RACE kit (ABI, Ambion) and 200 units of Superscript III reverse transcriptase (Invitrogen) at 55°C as described [3]. Four μl of cDNA were amplified by nested PCR in a 25-μl final volume containing Phusion DNA polymerase, GC Buffer (Fisher) and 0.4 μM of each primer. The outer primer for DUX4 cDNA amplification was: 5′-aggcgcaacctctcctagaaac-3′ and the inner primer was: 5′-tggaagcacccctcagcgaggaa-3′. Products were cloned and sequenced to confirm DUX4 mRNA amplification. For the restriction digest assay, 3’ RACE products (n = 4 injected legs per assay) were digested with BsaAI (New England Biolabs) overnight at 37°C. Samples were electrophoresed on a 1.5% agarose gel for 3 hrs and 100V and stained with ethidium bromide for UV visualization.

#### Western blot

Proteins were harvested from HEK293 cells transfected 2 days earlier with 2 micrograms of with the indicated plasmids. The DUX4-55aa construct was generated by recombinant PCR with details available upon request. A V5 tag was purposely not added to the 3’ end of the DUX4-55aa, thereby necessitating the use of DUX4-specific antibodies for western blot detection. All constructs (DUX4.V5, DUX4.HOX1.V5 and DUX4-55aa) were transcribed from the CMV promoter. Following separation of proteins on SDS-PAGE and transfer to PVDF, membranes were incubated with rabbit polyclonal anti-DUX4 primary antibodies (1:250; Biorybt), followed by HRP-coupled goat anti-rabbit secondary antibodies (1:100,000; Jackson Immunochemicals), and developed on film with chemiluminescence substrate (Pierce).

### In silico analyses

In silico analysis was performed by the Research Data and Computing Services (RDC) at the Research Institute at Nationwide Children’s Hospital. Lists of all human and mouse transcripts deposited in CCDS libraries were downloaded from the NCBI ftp server (ftp://ftp.ncbi.nlm.nih.gov/pub/CCDS). Stop codons were removed from each cDNA sequence and replaced with the V5 encoding sequences (5’ GGT AAG CCT ATC CCT AAC CCT CTC CTC GGT CTC GAT TCT AGC 3’) from Life Technologies/Invitrogen expression vectors. Sequences encompassing a consensus splice donor junction (5’ -AGGTAAGCCT—3’) and those with the “DUX4 site” (5’ -TGGTAAGCCT—3’) were compiled in S1 Table.

### Cell transfection and death assay

Caspase 3/7 activity was measured with the Apo-ONE Homogenous caspase-3/7 Assay (Promega, Madison, WI). HEK293 cells (65,000 cells/well) were transfected with 200ng of DNA and plated simultaneously on 96-well plates. Caspase 3/7 activity was measured 48 hours post-transfection using a fluorescent plate reader (SpectraMax M2, Molecular Devices, Sunnydale, CA.) Individual assays were performed in triplicate and reported as mean caspase activity relative to the samples that received DUX4.V5. Data reported are derived from two independent experiments.

## Supporting Information

S1 Table (XLSX)
